# Dentistry in the Forties

**Published:** 1892-12

**Authors:** L. C. Whiting

**Affiliations:** Saginaw, Mich.


### Dentistry in the Forties.

BY L. C. WHITING, SAGINAW, MICH.

Read before the Michigan State Dental Society.

The year 1840 marks a transition period in dentistry, from the use of ivory, animal and human teeth, to porcelain teeth on gold and silver plates. Previous to this time, a large portion of the artificial teeth used in the profession were manufactured from tusks of the sea horse. Human teeth and the teeth of animals were used for spaces requiring but one or two teeth. The successful dentist of this time was of necessity an artist, for he had not only to carve the blocks of sea-horse ivory into the shape of human teeth, but must also color them. The coloring was done with a hot iron. This work was sometimes so cleverly executed that it escaped ordinary observation, and was almost able to defy detection.

The principal defect of this ivory was its tendency to decay, and those wishing clean mouths and sweet breath required the constant aid of a dentist.

The usual way of keeping teeth in place was by gold bands attached to the natural teeth, or by pivoting them to roots. When but one or two teeth were required they were sometimes fastened by gold wire, but oftener by hickory pins, passing to cavities already existing or made for the purpose in the natural teeth. The natural teeth were pressed apart to set these in place. Full upper and lower blocks were held in position by spiral springs made of gold wire. These springs were used on gold plates for several years after porcelain teeth came into use.

Many full sets of porcelain teeth were inserted without plates. The teeth were held together by gold bands, or facings, and spiral springs were used to hold them in position in the mouth, imitating the sea-horse block work.

Parts of sets were made with gold facings and fastened to roots of teeth and to adjoining teeth with gold pivots and bands. The first set I ever made was of this kind, and I well remember the difficulty I had in cutting the band to fit the backs of the teeth. It was practically the same as our present bridge work, with the exception of the present improved fastening.

The durability of this work depended upon the condition of the roots. So many failures occurred that most dentists abandoned it and recommended extracting all roots and badly diseased teeth, and replacing with gold plates and teeth.

Dentists, as a rule, kept their improved methods a secret. I have paid one hundred dollars for half a day's instruction. Dr. Dodge, of New York City, took out a patent on atmospheric plates about the year 1844. The *American Dental Journal* was the first periodical in the interest of dentistry brought to my notice, and it was ably conducted. Amalgam was condemned, and no dentist used it who wished to stand well with the profession. Stockton also published a paper, which came out once a month.

The treatment of decayed teeth was very different from our present processes. Superficial decay was removed with the chisel and file. Large V-shaped spaces were made between the teeth, not only to remove decay but to prevent it. These spaces were called self-cleansing. When the decay was deep, the cavity was filled with gold or tin foil, but never built out, as at present. Lead was used to cap exposed nerves and gold put in over it. Front teeth, such as we now fill, when thought to be too much decayed to save, were cut off and pivot teeth inserted. Freshly-exposed nerves were taken out with a barbed wire and an artificial crown put on immediately. When the nerve was freshly exposed no future trouble was expected. When the nerve had died from exposure more care had to be taken, and some treatment was given to make them healthy before inserting the artificial substitute.

Transplanting teeth was one of the hobbies previous to the year 1840. Frequently persons would buy teeth of those in poorer circumstances, and have them transferred immediately to their own mouths. Serious cases of blood poisoning soon checked this branch of dental surgery.

Porcelain teeth were used in Paris some time before their introduction in this country. Billard, of Paris, had a world-wide reputation for making what was then called incorruptible teeth. Dentists in this country soon commenced manufacturing teeth and made improvements on them. Stockton, of Philadelphia, took the lead. Drs. Spooner and Allcock, of New York, and others, made teeth for their own use. Dr. Allcock was the first to arrange them in sets. Previous to this time, each dentist had to select and arrange his own as best he could, from a promiscuous collection. Every tooth had to be examined to see if the rivets were in the right place and otherwise perfect. Perfection was aimed at but not always attained. Human teeth had been freely used in the ordinary modes of dental work. After the battle of Waterloo twenty bushels of teeth are reported to have been extracted from the fallen soldiers for the use of dentists. Impressions of the mouth were taken in beeswax. Plaster impressions of the mouth were a great advance in the manufacture of artificial plates.

Previous to continuous gum work no practical method had been adopted for plumping out the mouth and face to its original shape, thus, in a measure removing the visible ravages of age and decay. Many were willing to pay a large price for artistic work in this line. Dr. John Allen, of Cincinnati, received five hundred dollars for a full upper and lower set of teeth with plumpers.

Later improvements in the dental art are so familiar to you all that they need no mention in this article. We take pride in our present achievements in filling teeth, but I doubt if we can boast of a greater percentage of success than the dentists of fifty years ago. Dr. Bancroft, of Almont, sends me some teeth which he says I filled for him previous to 1850. This was before any preparation of cohesive gold was used. The teeth are well preserved; although the fillings are soft, they accomplished what was aimed at, the preservation of the teeth. I have a tooth from my own mouth which was filled with amalgam, and as far as the filling was concerned did service for forty-five years. These fillings were put in as an experiment, and were made of filings from a Mexican dollar amalgamated with quick silver and made soft by the addition of tin foil. I believe it as good as any amalgam of the present time.

